# Management and control of communicable diseases in schools and other child care settings: systematic review on the incubation period and period of infectiousness

**DOI:** 10.1186/s12879-018-3095-8

**Published:** 2018-05-02

**Authors:** Ida Czumbel, Chantal Quinten, Pierluigi Lopalco, Jan C. Semenza, The ECDC expert panel working group, Alberto E. Tozzi, Catherine Weil-Oliver, Gordon Nichols, Hanne Nøkleby, Irina Brumboiu, Janneke Verheijen, Javier Segura del Pozo, Mira Kojouharova, Emese Szilágyi, Tiia Pertel

**Affiliations:** 1 European Centre for Disease Prevention and Control, Solna Municipality, Sweden; 2 Department of Translational Research and New Technologies in Medicine and Surgery, University of Pisa, Pisa, Italy

**Keywords:** Infectious diseases, Measles, Mumps, Rubella, Varicella, Pertussis

## Abstract

**Background:**

Information on the incubation period and period of infectiousness or shedding of infectious pathogens is critical for management and control of communicable diseases in schools and other childcare settings.

**Methods:**

We performed a systematic literature review (Pubmed and Embase) to identify and critically appraise all relevant published articles using incubation, infectiousness or shedding, and exclusion period as parameters for the search. No language, time, geographical or study design restrictions were applied.

**Results:**

A total of 112 articles met the eligibility criteria. A relatively large number were retrieved for gastrointestinal diseases and influenza or respiratory syncytial virus, but there were few or no studies for other diseases. Although a considerable number of publications reported the incubation and shedding periods, there was less evidence concerning the period of infectiousness. On average, five days of exclusion is considered for measles, mumps, rubella, varicella and pertussis. For other diseases, such as most cases of meningococcal disease, hepatitis A and influenza exclusion is considered as long as severe symptoms persist. However, these results are based on a diverse range of study characteristics, including age, treatment, vaccination, underlying diseases, diagnostic tools, viral load, study design and definitions, making statistical analysis difficult.

**Conclusions:**

Despite inconsistent definitions for key variables and the diversity of studies reviewed, published data provide sufficient quantitative estimates to inform decision making in schools and other childcare settings. The results can be used as a reference when deciding about the exclusion of a child with a communicable disease that both prevents exposure and avoids unnecessary absenteeism.

## Background

Illnesses caused by infectious diseases are common in children in schools and other childcare settings. Socio-economic factors can increase the risk of outbreaks among children and adolescents in these settings. Some infectious diseases are communicable, i.e. can be transmitted from one person to another, for example, via droplets, air suspensions, faeces, urine or skin-contact.

Optimal control of communicable diseases requires information on the incubation period and period of infectiousness, to inform operative measures such as temporary exclusion from the community to prevent further exposure [1, 2]. However, no systematic reviews exist that have thoroughly researched the available evidence on the incubation and infectiousness periods of communicable diseases in order to inform decisions about exclusion in schools and other childcare settings. One consequence of this is the lack of a common approach across European countries. Improved information about these parameters, as well as about the efficiency and cost aspects of exclusion policies, could contribute to more effective prevention and control of communicable diseases in schools and childcare settings and would be particularly useful for diseases that lack vaccination or treatment options [3, 4].

In view of this, the European Centre for Disease Prevention and Control (ECDC) commissioned a systematic review of the available evidence in the scientific literature in order to provide guidance on control of the communicable diseases in children and adolescents that account for the majority of disease outbreaks and absenteeism. The review also aimed to identify gaps in current knowledge about the epidemiology of infectious diseases and in particular to highlight diseases for which the required information is missing and areas where further research is needed [5].

Based on the results of the systematic review, this paper provides a comprehensive overview of the best available evidence and scientific knowledge on the incubation period, period of infectiousness or shedding of infectious pathogens, and the exclusion period for eight infectious diseases of public health importance in children aged 1 month to 18 years. The results are intended to help those responsible to define the minimum exclusion period from school or childcare setting for the duration of communicability of an infectious disease in order to limit disease spread and to avoid unnecessary long absenteeism.

## Methods

### Design and research questions

The research questions were:

- What is the incubation period of specified transmittable infectious diseases in children 1 month of age or older and teenagers?
- What is the period of infectiousness of specified transmittable diseases in children 1 month- 18 years of age or, if not available, what is the duration of shedding of specified transmittable diseases in children and teenagers?
- What is an appropriate setting-specific exclusion period for children and teenagers attending a school or childcare setting who are infected with specific transmittable diseases?

The diseases for the search were selected through repeated rounds of prioritisation taking into account transmissibility, severity and social concern of the proposed diseases.

The diseases prioritised for the search were:

- Measles; mumps; rubella; varicella; pertussis
- Meningococcal disease
- Enterovirus infections (non-polio, non- hand, foot, and mouth disease)
- Viral gastroenteritis (caused by adenovirus, astrovirus, noro−/calici−/sapovirus, rotavirus);
- Hepatitis A
- Salmonellosis (non-typhoid, typhoid, paratyphoid)
- Shigellosis;
- *Escherichia coli* infections
- Campylobacteriosis
- Giardiasis
- Airborne diseases (influenza, infectious mononucleosis, respiratory syncytial virus infections)
- Streptococcal infections (scarlet fever, streptococcal pharyngitis, impetigo)
- Other transmissible diseases of interest in children (roseola infantum, erythema infectiosum, staphylococcal impetigo, hospital colonisation by resistant pathogens and MRSA infections)

The final findings of the literature review were presented to the panel during meetings held at ECDC in November 2014 and February 2015 and their feedback was incorporated into the protocol for the final report [5].

### Search strategy and selection criteria

A systematic literature review was performed in PubMed up to August 2014 to identify available evidence on the incubation period, period of infectiousness or shedding, and exclusion period for the prioritised diseases for infants and adolescents only. However, because few results were generated, the scope of the search was expanded to also include Embase, the publication timeframe was extended up to June 2015 and the search criteria were enlarged to: A) the selected diseases, B) children aged from 1 month to 18 years, C) incubation period, D) period of infectiousness or shedding, E) setting-specific exclusion period.

No language, time, geographical restrictions were applied. The search included evidence from observational and experimental studies. Results were excluded for children with health characteristics that might affect the incubation period, and period of infectiousness or shedding.

We also included results from a search of other selected data sources [5] including the websites of the US Centers for Disease Control and Prevention (CDC) (http://www.cdc.gov) and the World Health Organization (WHO) (http://www.who.int/en/) as well as handbooks such as the American Academy of Paediatricians Red Book (2012), Managing infectious diseases in childcare and schools, a quick reference guide (2009), and a literature review from 2001 by Richardson et al.

### Data extraction and assessment

Data were extracted using pre-defined parameters: the description of the case definition; the definition of the incubation, infectiousness, duration of shedding and exclusion periods as the number of days from a defined point in time until another defined point in time, unless stated otherwise in the study; measures of variation, if available; and separate data for each infectious agent if more than one was involved. Additionally, the evidence tables included the identified quality limitations where applicable.

Other relevant data extracted related to: objectives; study design, period and duration; country and setting; source population, inclusion and exclusion criteria, sample size, age and gender; infectious agent, case definition, laboratory methods, outcome definition; results; comments; and the study author, journal and year of publication. More details about the data extraction and assessment can be found in the published literature research [5].

**Fig. 1:**
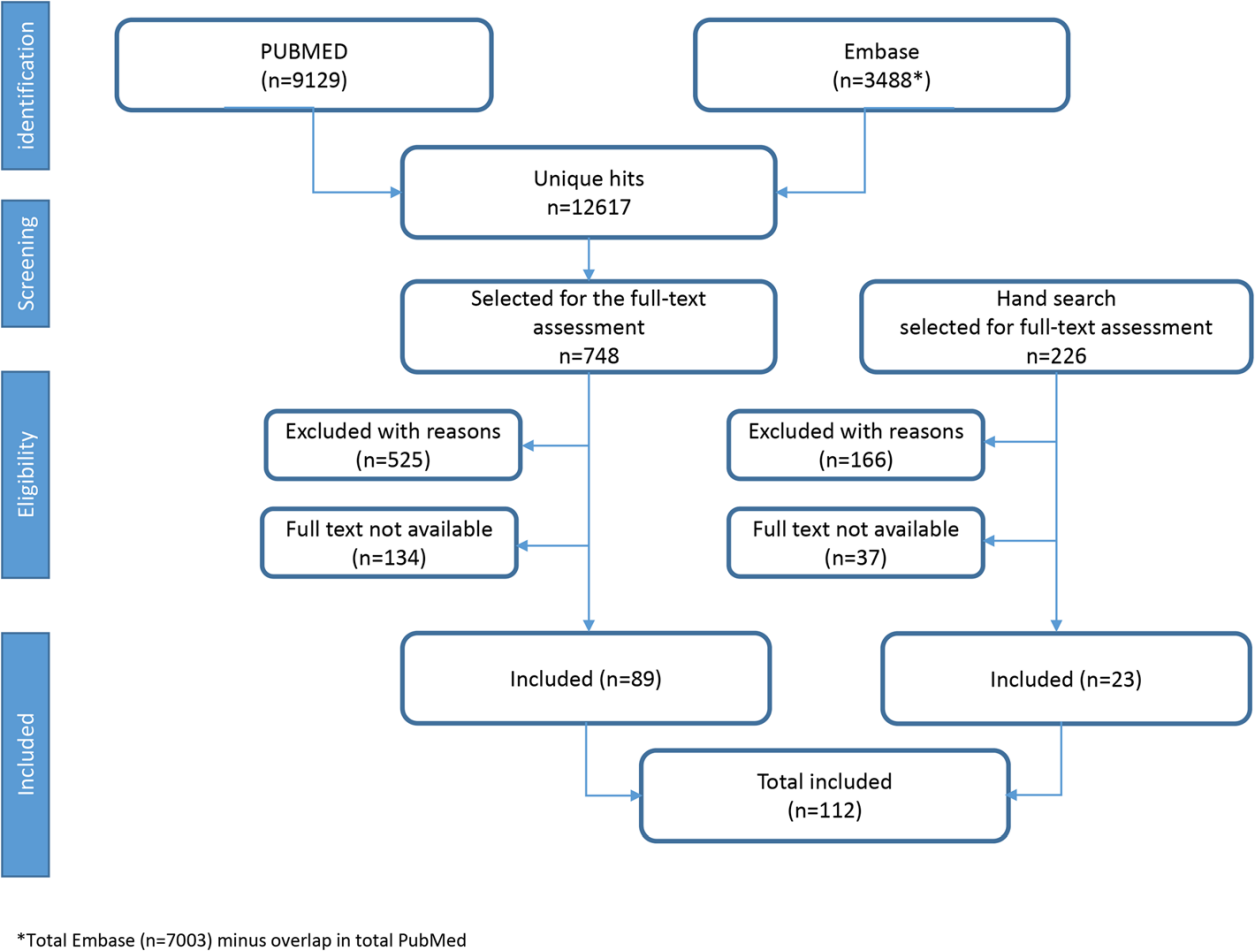
Flow diagram of the article selection process

As Fig. 1 shows, the search yielded 112 peer-reviewed articles that were eligible and met the inclusion criteria (974 were selected for full text assessment and of these, 171 could not be retrieved and 691 did not fulfil the inclusion criteria). The 112 eligible published articles were methodologically appraised by two reviewers based on Evidence Based Medicine checklists (see Appendix).^1^

## Results

This paper presents the results for eight selected diseases: measles, mumps, rubella, varicella, pertussis, meningococcal disease, hepatitis A and seasonal influenza.

Most evidence was found for measles, mumps, rubella, and varicella in terms of all the searched parameters, most likely due to the very specific symptom of onset of cutaneous eruption, considered as a key component to the clinical diagnosis of viral exanthemas.

### Measles

**Fig. 2:**
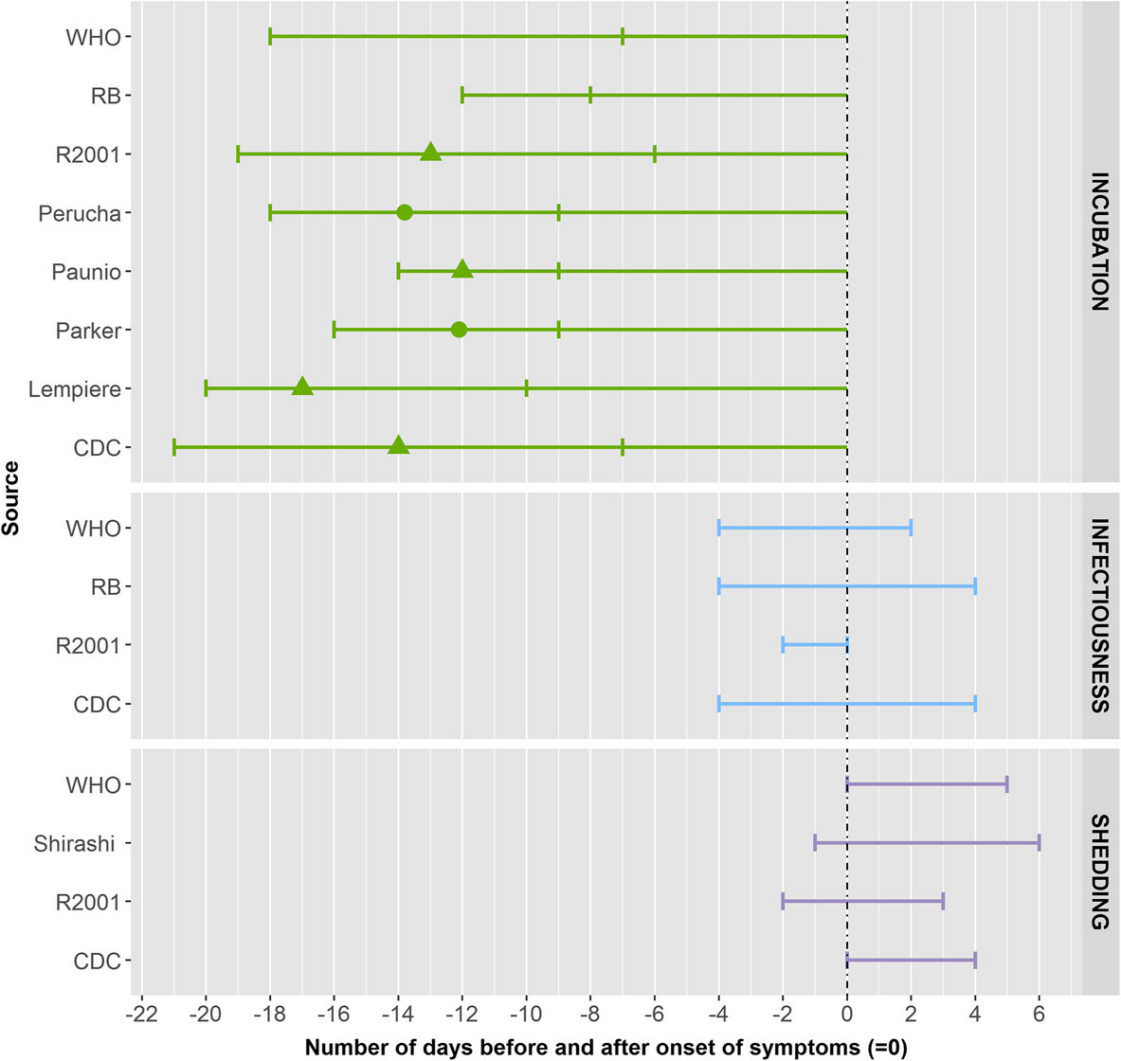
Summary measures for the incubation period, infectiousness and shedding period for measles by source. Legend: ▲: mean, ●: median, : minimum and maximum range, RB: Red Book, R2001: Richardson et al. (2001)

We identified seven eligible articles for measles. Most estimates arose from outbreak investigations carried out in different settings including schools [6–8], hospitals [9] and the community [10, 11]. In all but one article [10], the subjects were children aged 1 month of age or older and adolescents below 18 years of age. With respect to vaccination status, the subjects were vaccinated or had unknown vaccination status or had not been protected by previous exposure [6]. Five of the articles captured laboratory-confirmed cases using serology [7], PCR [8, 10], positive reverse-transcriptase PCR [11] or virus isolation in culture [9, 11] from serum, urine, nasopharyngeal exudate and respiratory secretion; laboratory methods were not described in two articles [6, 12]. We also included results from the search of other data sources (see above). (Fig. 2).

The incubation period ranged from between 6 and 21 days. Four articles showed a range of between 9 and 20 days, with a median value of around 13 days. In these four studies, the incubation period was defined as time from exposure to onset of rash or fever. The incubation period among those who had been vaccinated was found to be approximately 2 days shorter than that among those who had not been vaccinated in one study [7], but this observation would need to be confirmed by additional studies. No peer-reviewed articles on infectiousness were found. Other data sources described an infectiousness period of 4 days before and 4 days after the onset of rash. The duration of shedding ranged from between 2 days before to 6 days after the onset of rash [2]. In patients with immune or nutritional disorders the duration of shedding in respiratory secretions can be longer, up to 10 days from onset of fever [9]. Information on exclusion was available mainly in the grey literature. It states an exclusion of 4–5 days from onset of rash [13–15].

### Mumps

**Fig. 3:**
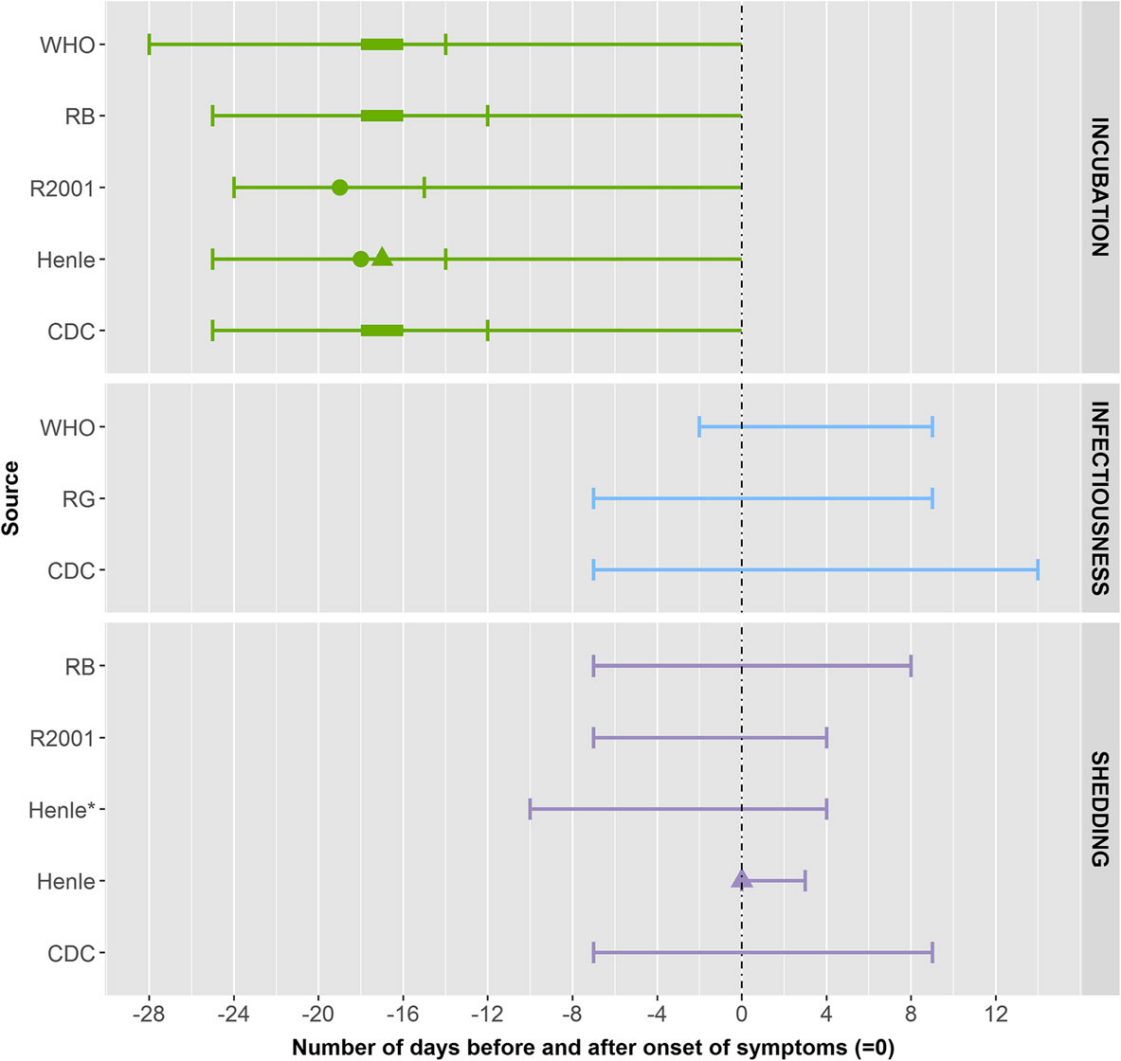
Summary measures for the incubation period, infectiousness and shedding period for mumps by source. Legend: ▲: mean, ●: median, ▬ interval [quantitative measure around the central tendency (mean or medium) or qualitative (usually) measure as provided by the authors], : minimum and maximum range, RB: Red Book, R2001: Richardson et al. (2001), RG: The 2009 ‘Managing infectious diseases in child care and school. A quick reference guide’

We identified two eligible peer-reviewed articles for mumps. The data are from an outbreak investigation among children aged between 16 months and 12 years [16] and from a case series analysis in a hospital setting; the age of children in the case series analysis was not reported [17] (Fig. 3).

The incubation period was defined as the time from exposure to the onset of symptoms (parotid swelling, submaxillary involvement or orchitis) and ranged from between 14 and 25 days. In other data sources the incubation period ranged from between 12 and 28 days, most often 16–18 days No data were found on infectiousness in peer-reviewed publications. In other data sources the longest period of infectiousness was found in CDC publications, which describe a range from between 7 days before to 11–14 days after parotitis onset [18]. Both peer-reviewed articles defined the duration of shedding as the period when mumps virus could be isolated both before and after onset of symptoms. In one of the articles, based on the virus isolated from the pharynx, the duration of shedding ranged from 2 days before to up to 5 days after the onset of parotitis [16]. In the second article, with salivary gland involvement, the period of shedding was found to be from 2 to 6 days prior to the onset of symptoms to 4 days after [17]. In patients with primary orchitis without any recognised involvement of the salivary gland the virus was isolated from saliva by mouth washing 10 days prior to the illness [17] up to 4 days after onset of symptoms. In other data sources, the duration of shedding ranged from between 7 days before to 9 days after onset of parotitis swelling [2, 13, 15]. Information on exclusion was found until 5 days of onset of parotitis [15, 19].

### Rubella

**Fig. 4:**
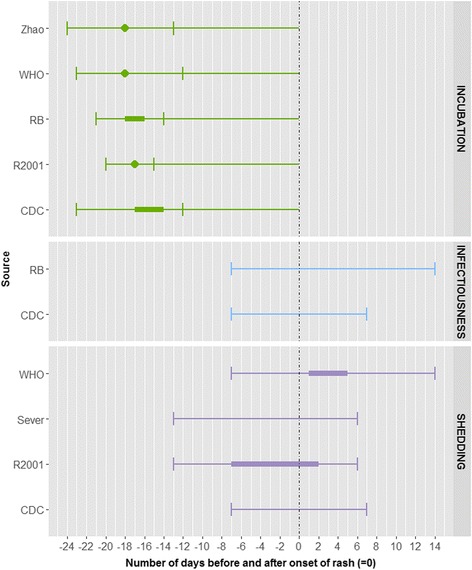
Summary measures for the incubation period, infectiousness and shedding period for rubella by source. Legend: ●: median, ▬ interval [quantitative measure around the central tendency (mean or medium) or qualitative (usually) measure as provided by the authors], : minimum and maximum range, RB: Red Book, R2001: Richardson et al. (2001)

We identified two eligible peer-reviewed articles. In both, data came from outbreak investigations [20, 21] in individuals less than 19 years old. The cases were defined based on clinical symptoms including fever, rash [21] and rubella-characteristic enlarged posterior auricular or sub-occipital lymph nodes [20]. Laboratory serological testing (ELISA) was used for detecting rubella-specific IgM, IgG [21]. Enterovirus interference method was used to detect viral RNA from throat swabs [20]. (Fig. 4).

The incubation period was defined as the time between exposure and the onset of any of the symptoms and ranged between 13 and 24 days [21]. In other data sources the incubation period ranged between 13 and 23 days, most often between 16 and 18 days.

Infectiousness was not reported in peer-reviewed articles. In the peer-reviewed articles, shedding was found as early as 13 days before the onset of rash and persisted for up to 6 days after onset [20], although in the majority of cases shedding was found 5 days before, and in all cases, 2 days before the onset of rash. CDC states that the disease is most infectious when a rash is erupting, but that the duration of shedding can be from 7 days before to 7 days after rash onset. In the WHO position paper rubella shedding was described as occurring between 7 days before up to 14 days after onset of rash, with maximal shedding occurring 1–5 days after rash [22]. Other data sources suggest an exclusion period of 5–6 days after onset of rash [15, 18, 22].

### Varicella

**Fig. 5:**
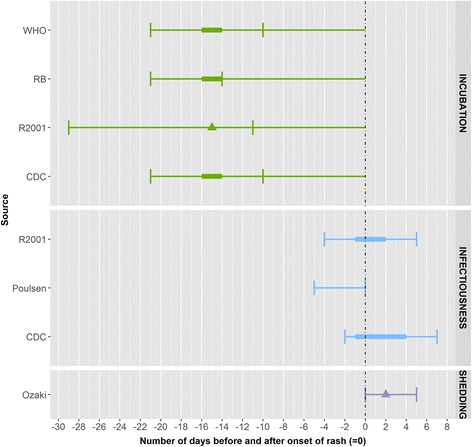
Summary measures for the incubation period, infectiousness and shedding period for varicella by source. Legend: ▲: mean, ▬ interval [quantitative measure around the central tendency (mean or medium) or qualitative (usually) measure as provided by the authors], : minimum and maximum range, RB: Red Book, R2001: Richardson et al. (2001)

We identified six eligible peer-reviewed articles on varicella. Data came from three outbreak investigations, two household studies and one case series analysis in children of different ages. Incubation period was not investigated in these studies. In other data sources the incubation period was found to be between 10 and 21 days with a mean/median of around 14–16 days depending on the contacts (Fig. 5).

Varicella is known to be infectious via contact certainly up to 5 days after the onset of symptoms, but may be longer and the infectiousness might persists by the end of the first week or the beginning of the second week of the eruption [23]. In Ozaki 1996 et al., the virus was isolated in cell culture in skin lesions in the first 5 days after the appearance of rash [24]. Two studies reporting on exclusion were conducted in school outbreaks where children were excluded from school for 7 days after the onset of symptoms or until all lesions were crusted [25, 26]. The exclusion seemed not to have been effective since most transmission already occurred after exposure to prodromal cases.

### Meningococcal disease

**Fig. 6:**
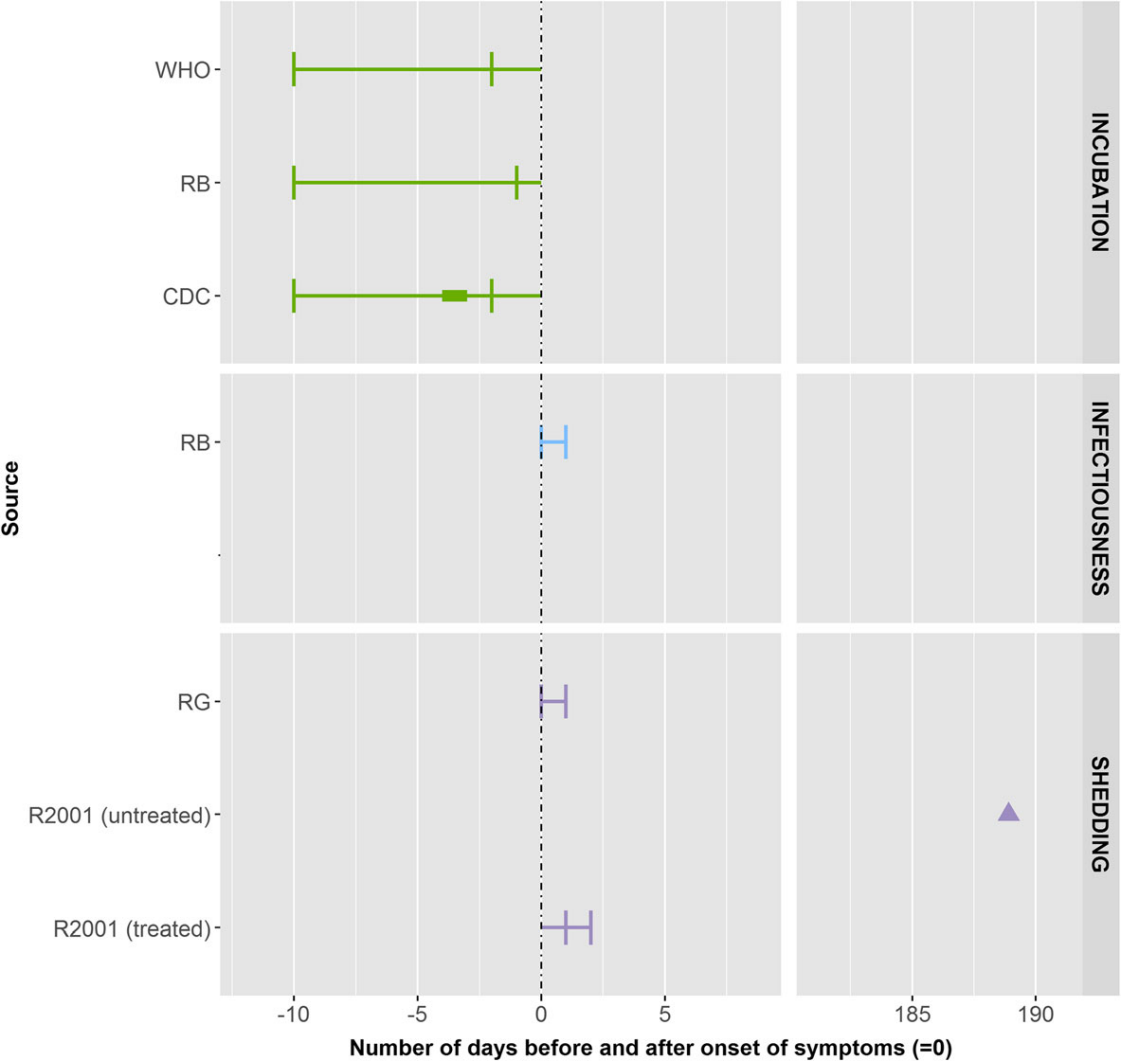
Summary measures for the incubation period, infectiousness and shedding period for meningitis by source. Legend: ▲: mean, ●: median, ▬ interval [quantitative measure around the central tendency (mean or medium) or qualitative (usually) measure as provided by the authors],  minimum and maximum range, RB: Red Book, R2001: Richardson et al. (2001), RG: The 2009 ‘Managing infectious diseases in childcare and school. A quick reference guide’

No eligible peer-reviewed articles were identified for meningococcal disease. In other data sources the incubation period ranged from between 1 and 10 days, most often between 1 and 4 days. Infectiousness and shedding were described as persisting for 1–2 days after the start of treatment [2, 13, 15]. In untreated patients the median duration of shedding was 9 months [2]. The literature revealed that the exclusion should start as soon as the disease is suspected and for at least 48 h from the start of treatment [15, 18, 19] (Fig. 6).

### Pertussis

**Fig. 7:**
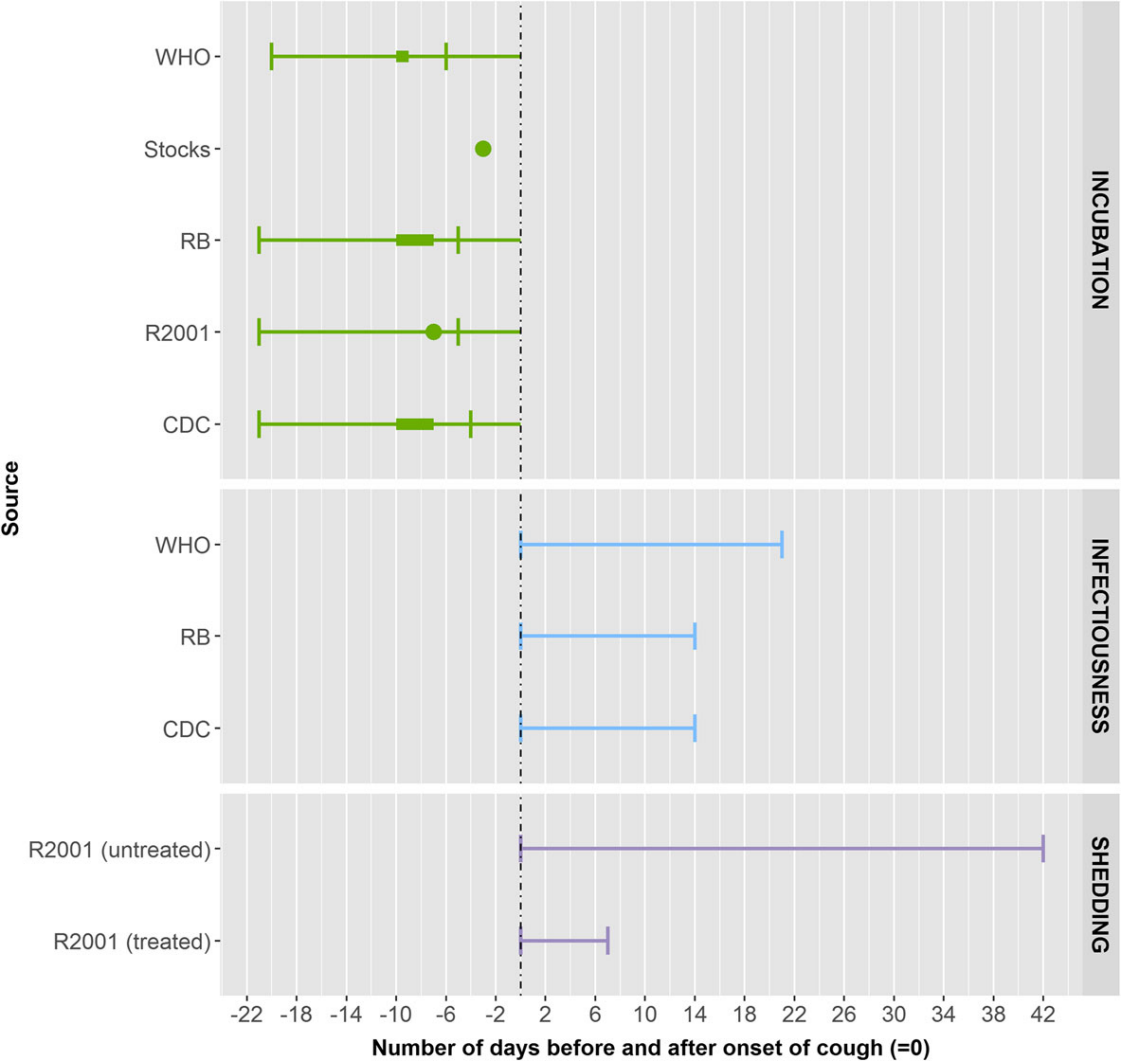
Summary measures for the incubation period, infectiousness and shedding period for pertussis by source. Legend: ●: median, ▬ interval [quantitative measure around the central tendency (mean or medium) or qualitative (usually) measure as provided by the authors],  minimum and maximum range, RB: Red Book, R2001: Richardson et al. (2001)

Two eligible peer-reviewed articles, one outbreak investigation and one descriptive study, were identified [27, 28]. The samples used for isolation of *Bordetella pertussis* were nasal swabs and sputum, using culture and identification by neutralisation or immunofluorescence test from notified cases. (Fig. 7).

The descriptive study showed an incubation period of 3 to 7 days with an unknown upper limit, based on speculations in light of recorded serial intervals [28]. In other data sources the incubation period ranged from between 4 and 21 days, usually 7–10 days [2, 13, 14]. The disease is described as most contagious in the first two weeks after cough onset [15].

In the outbreak investigation, the duration of shedding was measured as the isolation rate among all unvaccinated and untreated clinical cases over the study period and was up to 4 to 7 weeks after illness onset [27]. In other data sources the duration of shedding was found to be less than 7 days after onset of symptoms in those who were treated and 2–6 weeks in those who were untreated. The authors of the outbreak investigation study suggest that due to the long duration of shedding, exclusion from school for 3 weeks will not be effective [27]. When deciding on disease control measures, more attention should be paid to pre-school age contacts for whom the disease has more harmful consequences. In other data source, exclusion for pertussis for 5 days was described for patients receiving a full course of antimicrobial treatment [2, 13, 15, 19].

### Hepatitis a

**Fig. 8:**
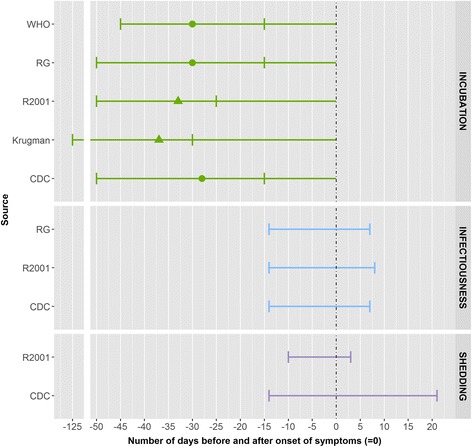
Summary measures for the incubation period, infectiousness and shedding period for hepatitis A by source. Legend: ▲: mean, ●: median, minimum and maximum range, RB: Red Book, R2001: Richardson et al. (2001), RG: The 2009 ‘Managing infectious diseases in child care and school. A quick reference guide’

We identified three peer-reviewed studies, two outbreak investigations and a study comparing epidemiological, clinical and immunological hepatitis A, conducted among school-age children in different school settings with one or more statements on the searched parameters. The laboratory results were confirmed using serum for IgM antibody to HAV, serum bilirubin, serum transaminase (SGOT) and bilirubin level in the urine [29–31]. (Fig. 8).

In Krugman et al., the incubation period was defined as the time between exposure and the first evidence of elevated serum transaminase activity (SGOT level above 100) [29]. In this particular study with a laboratory value as a reference for the incubation period the estimates were between 30 and 125 days, with a median of 37 days. It is important to note that this study was conducted among institutionalised children with low hygienic standards. Because of the laboratory value in the incubation period definition, asymptomatic cases were also included in the study. In Brodribb, the incubation period was defined as the time between exposure to the first case and onset of clinical symptoms (if any) in the wave of secondary cases [30] and the estimated period was 20–32 days. No data were available in the peer-reviewed articles on infectiousness and shedding periods.

In other data sources the estimates for the incubation period were up to 15–50 days with an average of 28–30 days. Most cases are infectious from 2 weeks before to 1 week after onset of symptoms [15, 19]. The estimates for duration of shedding ranged from between 1 and 2 weeks before to 1–3 weeks after onset of symptoms, with a peak just before the onset (usually dark urine) [15]. Exclusion from school until severe symptoms persist combined with application of hygienic measure was found useful [31] while the Red Book recommends one week of exclusion after onset of jaundice [13].

### Seasonal influenza

The search identified eight eligible peer-reviewed studies for influenza A or B. The laboratory results were obtained from nasal/pharyngeal washes or throat swabs using cell culture, fluorescent-antibody technique, cytopathic effect, hemadsorption, indirect immunofluorescence, hemagglutination inhibition testing or influenza virus rapid antigen detection.

**Fig. 9:**
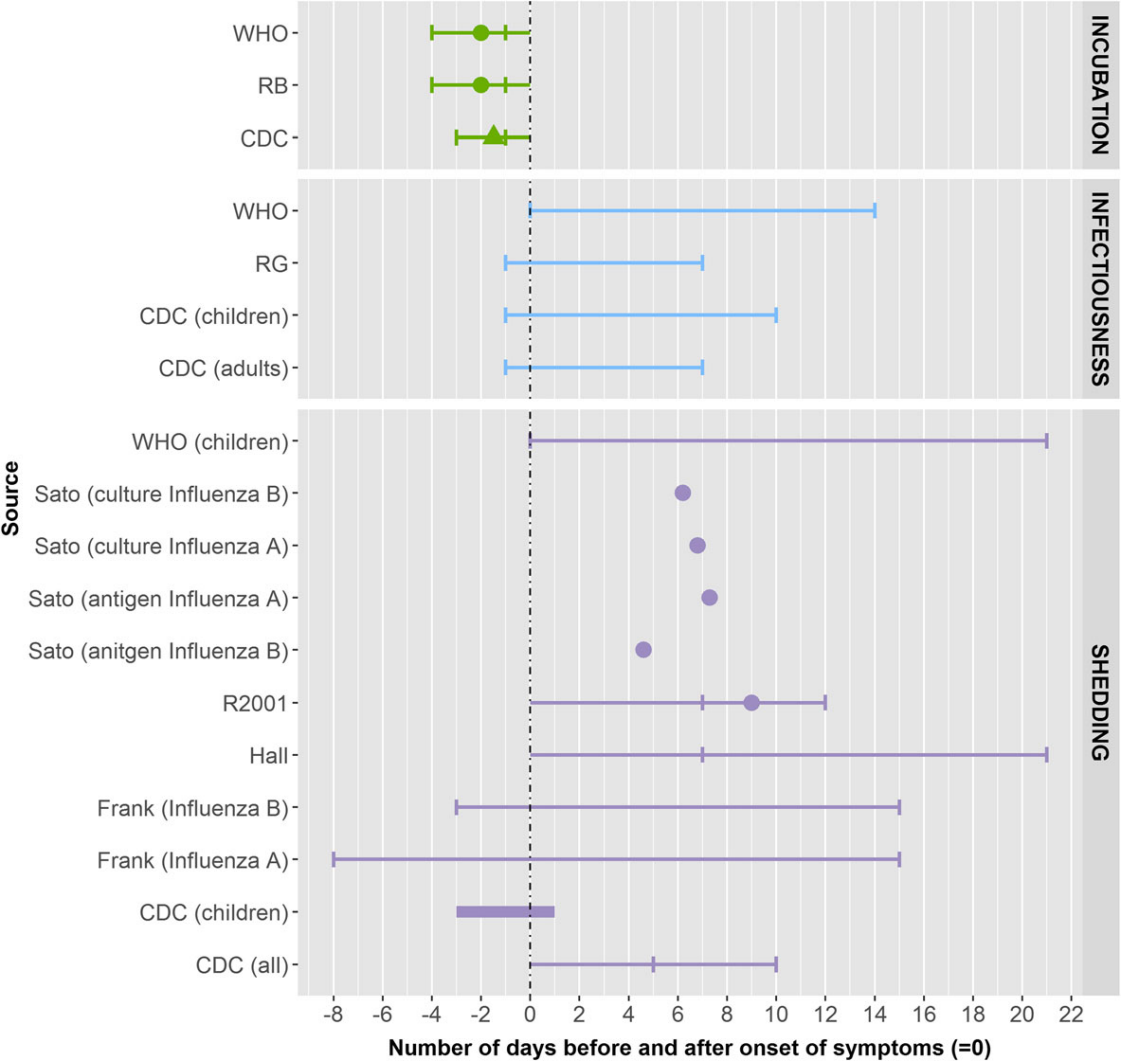
Summary measures for the incubation period, infectiousness and shedding period for influenza by source. Legend: ▲: mean, ●: median, ▬ interval [quantitative measure around the central tendency (mean or medium) or qualitative (usually) measure as provided by the authors],  minimum and maximum range, RB: Red Book, R2001: Richardson et al. (2001), RG: The 2009 ‘Managing infectious diseases in child care and school. A quick reference guide’

No peer-reviewed publications reported on the incubation period or period of infectiousness for influenza. In other data sources, an incubation period of 1–4 days is described, on average 2 days [2, 13, 19] and a period of infectiousness of from up to 1 day before to 10 days after onset of symptoms in children [15, 19] (Fig. 9).

Five peer-reviewed articles presented data on the duration of influenza A shedding, measured from onset of illness and or admission to hospital [32–36]. Three of these were case series analyses, one an outbreak investigation and one a retrospective follow-up study. A randomised controlled trial for measuring the efficacy of oseltamivir presented data on mean values but not on the entire period of shedding. The virus could be isolated as early as 8 days before the onset of symptoms and up to 15 days after onset [34]. A mean of around 7 days of shedding from onset of illness was reported for influenza A [32]. One study also reported on influenza B, and a mean of around 6 days measured by viral culture and 4.6 days measured by antigen detection was reported for influenza B [32].

In other data sources, shedding was reported to persist for up to 21 days in young children from the onset of illness [2, 15, 18, 19]. No studies reporting on the exclusion period were identified. According to one source, there is no need for exclusion unless the child is unable to participate in lessons [14].

## Discussion

In this review, we searched for and assessed available evidence on an important but neglected area of public health. Specifically, the review focused on the incubation period, period of infectiousness or shedding and exclusion period for eight infectious diseases that account for the majority of disease outbreaks and absenteeism among children and adolescents.

The key parameters originated from a comprehensive search in PubMed and Embase to identify published literature, complemented with estimates from other sources including WHO, CDC and clinical guidelines. We found that estimates obtained from these two types of sources did not differ substantially, although there were some discrepancies and outliers. Understanding the underlying disease mechanisms and the determinants of the incubation period are likely to be critical when interpreting these differences.

### Definition and measurement issues

The review highlighted some of the challenges in drawing conclusions from a diverse range of studies and difficulties in making comparisons based on diverse definitions and methods, as discussed below.

To compare the key parameters, it is critical to have clear definitions of the measurements. The exact time of exposure, the description of the symptoms used to define disease onset, the characteristics of the exposure, serotype, infective dose, the population characteristics, the study design and the diagnostic tools used for measurement can all have an impact on the value of these parameters [37]. In this context, outliers are most likely due to the lack of standardised methods and missing or varying definitions for measuring the estimates. Since there are no protocols in place related to research of the parameters of interest to this review, observations mostly rely on patients’ (or their parents’) recollection of the onset of symptoms or depend on local circumstances e.g., laboratory methods concerning the measurements.

The incubation period is defined as the time from infection to clinical onset. Therefore, when describing the incubation period, it is important that authors accurately define the symptom of reference and the date of onset of the symptom. This is because some diseases might have more than one symptom of onset e.g., fever, rash or coughing, with different timing, which would result in a different duration of the incubation period. Inconsistent description of the symptoms used as the onset reference would also result in a different duration of the incubation period. It can also be challenging to define the incubation period when the exact time of exposure is unknown or where the accurate recording of symptoms is difficult e.g., asymptomatic hepatitis A or unspecific symptoms of onset or when the timing of symptoms onset relies on good recall by patients. The inclusion of asymptomatic cases or in some contexts, low levels of exposure with poor hygiene standards could have prolonged the length of the incubation period [29]. When the incubation period was not available for diseases, serial intervals were retrieved [28, 38]. For highly infectious diseases such as measles or diseases with asymptomatic onset such as hepatitis A in settings with frequent contact between subjects, serial interval is likely to be a good approximation for the incubation period [30].

Differences might also exist for other key parameters. It was difficult to compare periods of infectiousness for the diseases of interest because we found very limited evidence on this in the peer-reviewed literature. Pathogen shedding and infectiousness are closely related – mostly the period of infectiousness is based on shedding or viral excretion data [39] – so, for some diseases, infectiousness could perhaps be determined from data on shedding. However, the results are highly influenced by the sampling methods, the frequency of sampling, the specimen and the laboratory method used as well as by the definition of the parameters for the period of shedding.

For instance, the value of the duration of shedding depends on the point at which measuring starts, e.g. at the first visit to the clinic or at the start of treatment. We identified articles where the measurement of the duration of shedding only started at the time of hospital admission [33, 40–43], although it is important to note that these studies mainly focused on the effect of treatment.

With respect to the impact of laboratory methods used, one example of the consequences for estimating the period of infectiousness is the shorter duration of virus excretion as measured by viral culture for influenza as compared to measurement by reverse transcription polymerase chain reaction (RT-PCR). When interpreting the estimation for infectiousness or shedding, the diagnostic methods used need to be taken into account.

Shedding before symptoms appear seems to be independent of sub-type, age or antiviral therapy [44]. For instance, oseltamivir treatment was not associated with statistically significant reduction in the duration of viral shedding in influenza patients [45]. Further, excretion may occur after recovery or in asymptomatic carriers. In terms of underlying diseases, prolonged shedding could be found in children, immuno-compromised individuals, and patients with underlying diseases including those receiving corticosteroid or other immunotherapy agents [1].

### Exclusion issues

Presenting conclusive data on exclusion is difficult because measures may be influenced by a range of factors, such as the age of the affected child, the setting and staff availability [46]. The decision to exclude a child largely depends on the perceived severity of the condition and its potential impact on the health of the affected child, other children and adolescents, and the wider community, and cannot therefore be completely evidence-based. Such decisions also need to consider the fitness of affected child to attend lessons and the ability of staff to care for the child and for other children.

Decisions about the length of the exclusion period should be based on data on infectiousness if they exist or, if not, on data on shedding. The availability of immunological and molecular methods has brought new perspectives to this area of research because of the high speed and quantity of data generation [47]. Antigen ELISA, latex agglutination and immune-chromatography are the methods used nowadays to detect infectious virion/viral antigen. RT-PCR detection of viruses present in immune complexes can happen, but it does not necessarily mean the presence of infectious virion. Thus, when taking decisions about exclusion based on the period of shedding, the impact of different laboratory methods used to detect the shedding of virus or bacteria should always be considered.

The need for exclusion should be considered carefully. For some infectious diseases, even where there is evidence of shedding, the risk of transmission could be relatively low.

In the case of viral skin exanthema, this can be infectious before children develop a clinical illness [9, 17, 23] and exclusion might, therefore, be not fully effective. The child’s ability to participate in lessons as well as relevant socio-economic factors should also be taken into account when taking the decision on the exclusion. For immuno-compromised patients exclusion should be considered for the whole duration of illness. Another important aspect to be considered is high-risk close contacts, such as pregnant mothers, younger siblings or immuno-compromised relatives.

For some diseases, available recommendations on exclusion practice differ. Children with hepatitis A could be infectious 2 weeks before the onset of unspecific symptoms and infectiousness diminishes rapidly after symptoms appear. For this reason, the exclusion is usually recommended until clinical recovery. by Reid et al. [31] Exclusion has been specifically recommended for younger (< 5 years) children by Richardson et al. [2] and for those who are unable to maintain good personal hygiene by Krugman et al. [29] . Some authors deem exclusion to not be necessary, due to the mildness of symptoms, and recommend that standard hygienic measures should be applied during the whole course of the infections [48, 49]. However, CDC recommends one week of exclusion after onset of symptoms, when this is defined as jaundice. It is also important to note that asymptomatic cases of hepatitis A could contribute to further transmission.

Another important finding is the lack of evidence of the effectiveness of exclusion. Information regarding the exclusion period for a child with any of these infectious diseases was rarely discussed in the peer-reviewed literature and, when it was discussed, it was mostly in the context of contacts exclusion or school closure which were not the focus of our review. These limitations indicate areas for future research, including epidemiological research and evaluation of the effectiveness of exclusion policies, although the latter would require the development of standardised approaches to measurement of exclusion effectiveness. The results of this review can be further strengthened by applying stringent methodological standards such as experimental study designs to test the public health benefits of school exclusion in relation to the incubation period, period of infectiousness and shedding. Insights from such studies could be incorporated into updated guidance in the future.

## Conclusions

This review summarizes the current knowledge of the best available evidence from the scientific literature regarding the incubation period, shedding, and infectiousness of specific communicable diseases. We present the minimum and maximum time interval for the key parameters identified by the search. In addition to the above values, information on the most common values (mean or median, if reported) can be summarized as follows: The incubation period for measles ranged between 6 and 21 days with a median around 13 days; for mumps and rubella the most common value ranged between 16 and 18 days; and for varicella the mean/median was around 14–16 days, depending on the contacts. The incubation period for pertussis was described as usually 7 to 10 days. Incubation period for meningococcal disease was usually less than 4 days and for influenza a median of 2 days was found within a range of 1 and 4 days. A median of 37 days was reported based on serum levels for hepatitis A. Considering that many aspects play a role in the decision for which diseases to exclude and for how long, such as severity of the disease, immune status, socio-economic burden, feasibility and parental considerations, infectiousness data can be added to the methodological information when defining the minimum temporary exclusion from school or other childcare settings. Concluding on our findings on infectiousness, the measles virus was reported to be isolated 4 days before until 4 days after onset, however a median or range for infectiousness was not reported. Mumps was found contagious at the greatest extent 2 days before, up to 5 days of onset of parotitis, although the virus could be isolated up to 14 days after parotitis onset. Although in some studies rubella virus was isolated from 7 days before until 14 days after onset, the period when those infected were most contagious extends from a few (2 days) days before to 7 days after onset of a rash. Varicella was found most contagious while the rash is spreading until the lesions have crusted over, certainly up to 5 days after onset of the rash.

Meningococcal disease infectiousness was persisting up to 1 and 2 days after effective treatment. For pertussis shedding was reported up to seven days after onset of cough, if treated.

Influenza was found to be most contagious 1 day before until 7 days after onset, but can be longer in children. Hepatitis virus was reported most infectious 2 weeks before and 8 days after onset of illness.

Our searched revealed exclusion periods for 4 to 5 days for measles, 5 days for mumps and pertussis, 5 to 7 days for rubella, and 5 to 6 days for varicella. For influenza the search did not report recommendations on exclusion, unless the child is unable to participate in lessons. For meningococcal disease exclusion should start as soon as the disease is suspected, and criteria will be applied by the severity of the disease and for at least 48 h from the start of treatment. For hepatitis A, sources recommend one week of exclusion after onset of jaundice.

These findings demonstrate the strengths and weaknesses of the current knowledge base, a topic which is often encountered in clinical settings. The results can be used as a reference point for decision making on the exclusion of a child with a communicable disease to prevent exposure and avoid unnecessary long absenteeism in schools or other childcare settings and might call for a review of some local, regional, or national recommendations.

## Appendix

**Table 1:** CoCanCPG adapted checklist for critical appraisal of literature

Author, year	Ida Czumbel, Chantal Quinten, Pierluigi Lopalco, Jan C. Semenza 2018
Journal	BMC infectious diseases
Internal validity	
The study addresses a clearly focused question	Page 4
The study population is clearly described	Page 4
The population is representative of the source population	Extraction table: https://www.ecdc.europa.eu/en/publications-data/extraction-tables-systematic-review-incubation-and-infectiousnessshedding-period
Cases are clearly defined	Extraction table: https://www.ecdc.europa.eu/en/publications-data/extraction-tables-systematic-review-incubation-and-infectiousnessshedding-period
Pathogen presence is lab-confirmed via standard valid and reliable methods (in outbreak investigations: at least once, in other studies: for most cases)(not necessary in case of erythematous diseases)	Extraction table: https://www.ecdc.europa.eu/en/publications-data/extraction-tables-systematic-review-incubation-and-infectiosnessshedding-period
The outcomes are clearly defined	Pages 6–11
Where applicable, sampling frequency is sufficient	Extraction table: https://www.ecdc.europa.eu/en/publications-data/extraction-tables-systematic-review-incubation-and-infectiousnessshedding-period
Where applicable, modifying variables (such as age, treatment, vaccination status, symptoms) are identified and taken into account in the analysis	Extraction table: https://www.ecdc.europa.eu/en/publications-data/extraction-tables-systematic-review-incubation-and-infectiousnessshedding-period
Variation (e.g. range, SD) in outcome of interest is provided	Pages 6–11
Where applicable, variation (e.g. range, SD) in outcome of interest is provided for separate strata	Pages 6–11
The outcome of interest the main subject of the paper	Pages 6–11
External validity	
The population is representative of the target population (otherwise healthy children in day care facilities or schools)	Extraction table: https://www.ecdc.europa.eu/en/publications-data/extraction-tables-systematic-review-incubation-and-infectiousnessshedding-period
Overall assessment of the study	
Are the results valid?	Level of evidence Source of data (Adapted Pallas) I. Systematic review, metaanalysis or well-designed epidemilogic or experimental study with ≥50 subjects II. Well-designed epidemilogic or experimental study with 5–50 subjects III. Case reports with < 5 subjects, or poorly substantiated larger study IV. Opinion or clinical experience of experts (not supported by published data) Level (I) A evidence: varicella Level (II) B evidence: measles, mumps, rubella, pertussis, meningococcal disease, EHEC, hepatitis A, influenza,
Design-specific comments/limitations (e.g. in case of trials)	for certain diseases only a few studies exist difficulty in finding the relevant information in a systematic search, as parameters are not always in title/abstract/key words poor sampling procedures, poor definition of key variables, poor reporting of study population and small sample size no standards on the effectiveness of public health interventions e.g. exclusions exist thus no conclusions on the effectiveness of school exclusion can be drawn based on our findings
General comments/limitation	Relevant publications in the field of infectious diseases also include outbreak investigations, surveillance studies or other observational studies and for these studies no standard CoCanCPG checklists are available, for the existing review the checklist was adapted.
General comment	The findings could serve as a basis for the development of an evidence based document on minimum school leave for an infectious disease

## Abbreviations

CDC: USA Centre for Disease Prevention and Control
ECDC: European Centre for Disease Prevention and Control
ELISA: Enzyme-linked Immunosorbent Assay
Embase: Biomedical and pharmacological database of published literature
PCR: Polymerase Chain Reaction
Quick Reference Guide: Managing infectious diseases in child care and schools: a quick reference guide
RB: American Academy Paediatricians Red Book
RNA: Ribonucleic Acid
RT-PCR: Reverse-transcription Polymerase Chain Reaction
SGOT: Blood (Serum) Glutamic-Oxaloacetic Transaminase
VPD: Vaccine Preventable Diseases
WHO: World Health Organization

## Footnotes

1 The Coordination of Cancer Clinical Practice Guidelines (CoCanCPG, http://www.cocancpg.eu/) checklists that were used for this review are a combination of the PRISMA (http://www.prisma-statement.org) and Strobe Guidelines (http://www.strobe-statement.org/) on the most important criteria on publication quality. Existing CoCanCPG checklists have been adapted in order to apply to outbreak investigations, surveillance studies or other observational studies too, since standard CoCanCPG checklists are not available for these studies.
